# Draft Genome Sequences of Three Isolates of *Golubevia* sp. Basidiomycete Fungi Isolated from Powdery Mildew Pustules

**DOI:** 10.1128/MRA.00473-20

**Published:** 2020-06-04

**Authors:** Lina Russ, Sven Warris, Carin Lombaers-van der Plas, Lia H. Groenenboom-de Haas, Jan H. G. Cordewener, Elio Schijlen, Jürgen Köhl

**Affiliations:** a Wageningen Plant Research, Business Unit Biointeractions & Plant Health, Wageningen University & Research, Wageningen, Netherlands; b Wageningen Plant Research, Business Unit Bioscience, Wageningen University & Research, Wageningen, Netherlands; Broad Institute

## Abstract

The genomes of three *Golubevia* isolates (BC0812, BC0850, and BC0902) that have been shown to reduce conidiation of Blumeria graminis f. sp. *tritici* were sequenced using a dual-platform approach. The assembled genomes will help to elucidate the molecular mechanisms underlying the biocontrol effect of this understudied group.

## ANNOUNCEMENT

A recent screening for potential antagonists against the causal agent of powdery mildew (Blumeria graminis f. sp. *tritici*) in common wheat (Triticum aestivum) identified three fungal isolates to significantly reduce conidiation of the pathogen (1). Phylogenetic analysis placed the isolates into the new genus *Golubevia*, formerly *Tilletiopsis* (2). Isolates of this genus of anamorphic yeasts reduced powdery mildew diseases in barley, rose, and cucumber (3–5).

Isolates were collected from mildew pustules according to Köhl et al. (1). Fresh mycelium was harvested from oatmeal agar and added to 50 μl extraction buffer (10 mM Tris-HCl [pH 7.4], 0.65% sodium sulfite, 1.5% polyvinylpyrrolidone [PVP]). Biomass was ground by alternating bead-beating for 4 × 20 s (25 beads/s) with snap-freezing. DNA was isolated with the Gentra PureGene Yeast/Bact. kit (Qiagen) by incubating the sample in cell lysis buffer with 45 μg proteinase K at 55°C for 1 h. The following steps were performed according to the protocol.

Barcoded SMRTbell libraries were prepared by subjecting the DNA of individual isolates to damage repair (NEBNext formalin-fixed, paraffin-embedded [FFPE] repair mix) and g-TUBE shearing (Covaris) to an approximately 10-kb peak fragment size. After ligation of the barcoded adapters, the samples were pooled to 12-plex and subjected to ExoVII treatment. The pooled SMRTbell libraries were used for subsequent damage repair and AMPure XP (Agencourt) cleanup. The final libraries were quantified using a Qubit fluorometer (Thermo Fisher). The library size was assessed using a fragment analyzer (Agilent). SMRTbell DNA polymerase binding complexes (v2.0) were loaded by diffusion (sequencing chemistry 2.1, 5 to 10 pM loading) and sequenced for 10 h per cell after 120 min immobilization on a Sequel system (PacBio). This resulted in totals of 93,534 (BC0812), 375,003 (BC0850), and 393,769 (BC0902) subreads with average lengths of 4,054 bp, 3,542 bp, and 3,053 bp, respectively. The subreads were demultiplexed by barcode alignment using pyPaSWAS v3.0 (https://github.com/swarris/pyPaSWAS/wiki/Demultiplexing) (6).

The libraries for Illumina sequencing were constructed from randomly sheared DNA fragments (Covaris E210) aiming for a 300- to 500-bp insert size using the NEXTflex ChIP-seq kit (Bioo Scientific). The equimolar library pool was loaded onto an Illumina paired-end flow cell for cluster generation using a cBot (Illumina) and sequenced on an Illumina HiSeq 2500 instrument using 125, 7, and 125 flow cycles for forward, index, and reverse reads, respectively. This resulted in a total of 26,820,332 (BC0812), 36,508,062 (BC0850), and 24,040,242 (BC0902) paired-end reads that were quality checked using FastQC v0.11.7 (7).

Hybrid assemblies were generated from demultiplexed PacBio subreads and Illumina reads using SPAdes v3.11.1 (k-mers 21, 33, 55, 77, 99, and 111) (8). The results are summarized in Table 1.

**TABLE 1:** Characteristics and accession numbers of the genomes of *Golubevia* sp. isolates

Isolate	Genome size (bp)	Genome coverage^*a*^ (×)	No. of contigs ≥500 bp	*N*_50_ (bp)	Longest contig (bp)	Total no. of genes	GC content (%)	BUSCO score (%)	GenBank accession no.	SRA accession no.
BC0812	25,820,737	171	160	508,978	1,317,127	8,525	44.95	97.7	JAAVVH000000000	SRX6947161,^*b*^ SRX6947158^*c*^
BC0850	25,851,279	264	89	871,951	2,230,207	8,566	44.95	98.1	JAAVVG000000000	SRX6947160,^*b*^ SRX6947157^*c*^
BC0902	25,811,735	187	82	742,457	2,038,960	8,612	44.97	98.0	JAAVVI000000000	SRX6947162,^*b*^ SRX6947159^*c*^

a Combined PacBio/Illumina libraries used as input for assembly.

b PacBio reads.

c Illumina reads.

Metatranscriptomic reads from *in planta* bioassays with the *Golubevia* strains (9) were mapped to the assemblies with HISAT2 (10). The mapped reads were fed into the BRAKER1 pipeline for gene prediction (11). This resulted in identification of 8,525 (BC0812), 8,566 (BC0850), and 8,612 (BC0902) genes that were functionally annotated with Blast2GO (BioBam). The completeness of the annotation was estimated using the single ortholog database basidiomycota_odb9 (BUSCO v3), resulting in scores above 97% (Table 1) (12).

### Data availability.

This whole-genome shotgun project has been deposited in DDBJ/ENA/GenBank under the accession numbers JAAVVG000000000 (BC0850), JAAVVH000000000 (BC0812), and JAAVVI000000000 (BC0902) and BioProject accession number PRJNA575625.
